# A modified swan incision combined with lateral rectus suspension for exotropia: a retrospective cohort study demonstrating shorter operative time and faster recovery

**DOI:** 10.3389/fmed.2025.1571642

**Published:** 2025-09-19

**Authors:** Jiao Liu, Rongjie Guo, Wenhao Shen, Jiaxuan Jiang, Yiran Chu, Changyu Wu, Kai Hu

**Affiliations:** Department of Ophthalmology, Nanjing Drum Tower Hospital, Affiliated Hospital of Medical School, Nanjing University, Nanjing, China

**Keywords:** exotropia, modified swan incision, suspension, inflammation, strabismus surgery

## Abstract

**Background:**

A modified Swan incision (MSI) combined with lateral rectus muscle suspension surgery represents an innovative strabismus surgical technique developed by our research team. Compared to the conventional Swan incision, the MSI features a 50% reduction in length with more posterior placement. The suspension technique is a modified recession approach that corrects strabismus by creating a hammock-like structure to reposition the muscle at new insertion site, rather than directly suturing the muscle to the sclera. This study aims to evaluate the safety, efficacy, and potential advantages of this MSI technique for exotropia.

**Methods:**

A retrospective cohort study was conducted on 66 patients with exotropia treated from January 2024 to December 2024. The patients were divided into two groups based on actual surgical procedures: the MSI group (33 patients, 42 eyes) and the control group (33 patients, 36 eyes). The MSI group underwent the MSI technique, while the control group underwent traditional Parks incision combined with lateral rectus recession. The surgical time, postoperative recovery, and correction outcomes were compared between the two groups.

**Results:**

The MSI group showed significantly shorter surgical time (*P* < 0.0001) and lower redness score at 1 week postoperatively (*P* < 0.001), with comparable surgical success rate to control group (*P* > 0.9999).

**Conclusion:**

The MSI combined with lateral rectus suspension surgery demonstrates both efficacy in ocular alignment correction and favorable safety in surgery. This technique significantly shortens surgical time, reduces tissue damage, improves postoperative recovery, making it a valuable option for widespread application.

## 1 Introduction

**FIGURE 1:**
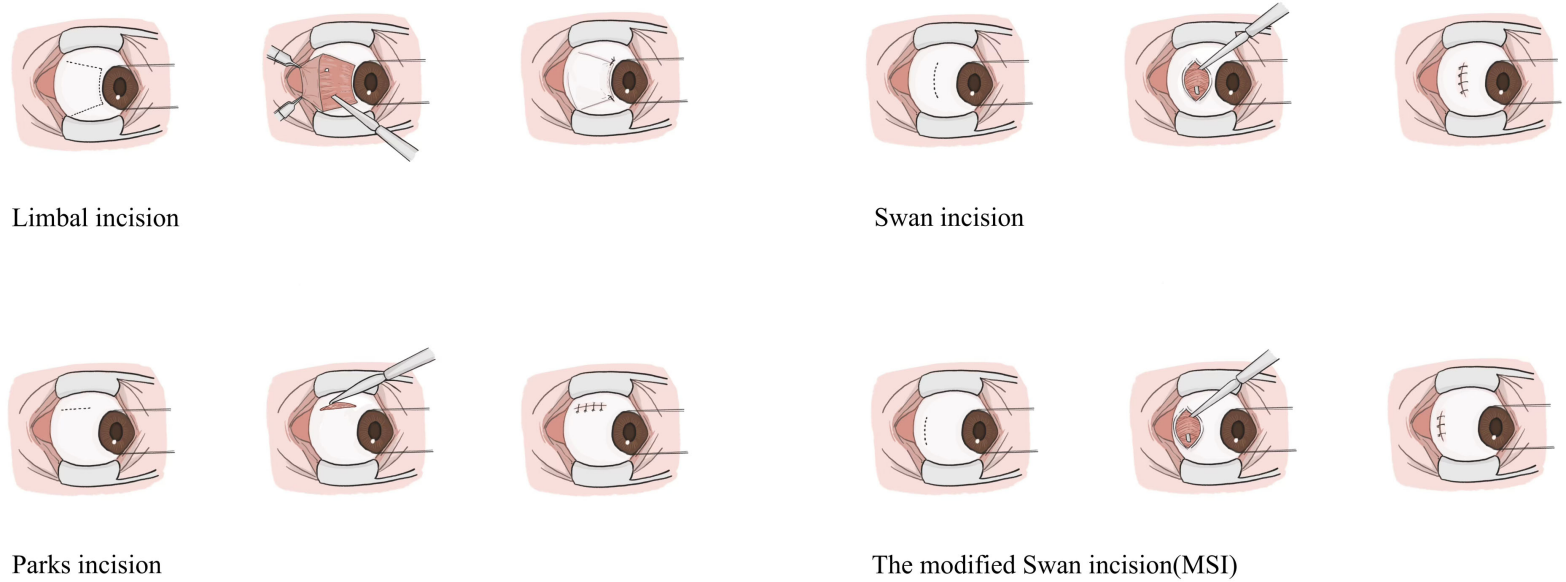
Schematic representation of several common conjunctival incisions in strabismus surgery.

Exotropia refers to the outward deviation of the eyes and can be classified into several types: pseudoexotropia, exophoria, intermittent exotropia, constant exotropia, and convergence insufficiency (1). For cases of intermittent exotropia, constant exotropia accompanied by amblyopia or a large angle of deviation, surgery is the primary clinical treatment approach (2, 3). In strabismus surgery, the type and location of the conjunctival incisions depend on the number and type of muscles operated on and the surgery technique. Currently, the most commonly employed traditional incision techniques in strabismus surgery include the rectus muscle insertion incision (Swan incision), limbal incision, and fornix conjunctival incision (Parks incision) (4, 5), as shown in Figure 1. In addition to these established methods, numerous modified incision techniques have been developed, enhancing surgical precision and outcomes. These include a modified “cross” Parks incision, L-shaped incisions, a conjunctival incision extending from the 2 o’clock to the 10 o’clock position, and minimally invasive strabismus surgery (MISS) incisions, which feature parallel cuts on either side of the rectus muscle (6–10).

The limbal incision can expose the muscles, avoid damage to the muscles, and allow multiple muscles to be operated simultaneously. However, this type of incision can cause extensive tissue damage and intense discomforts for patients after surgery, as well as damage to limbal stem cells (10, 11). Parks popularized the fornix conjunctival incision which remains covered by the eyelids and decreases the postoperative discomfort significantly. Compared with the limbal incision, the fornix conjunctival incision displays no visible reduction in the area of anatomical disruption between the muscle and peri muscular tissue and requires more skill of the surgeon (11). The paralimbic incision, or “Swan incision”, places the conjunctival incision parallel to the limbus and behind the muscle insertion, which can expose the muscles clearly but easily damage the muscles and result in tissue adhesions. Besides the Swan incision will affect the appearance because of the exposure to the palpebral fissure area (6).

The MSI is an improvement upon the traditional Swan incision. It is made when the eye is in primary position (refers to the position when both eyes are looking straight ahead), located within 2 mm of the medial and lateral canthi, and approximately 5 mm in length, perpendicular to the medial or lateral rectus. Specifically, the temporal MSI incision is about 10 mm posterior to the temporal corneal margin, and the nasal MSI incision is about 8 mm posterior to the nasal corneal margin. The exact measurements may vary slightly for different individuals.

**FIGURE 2:**
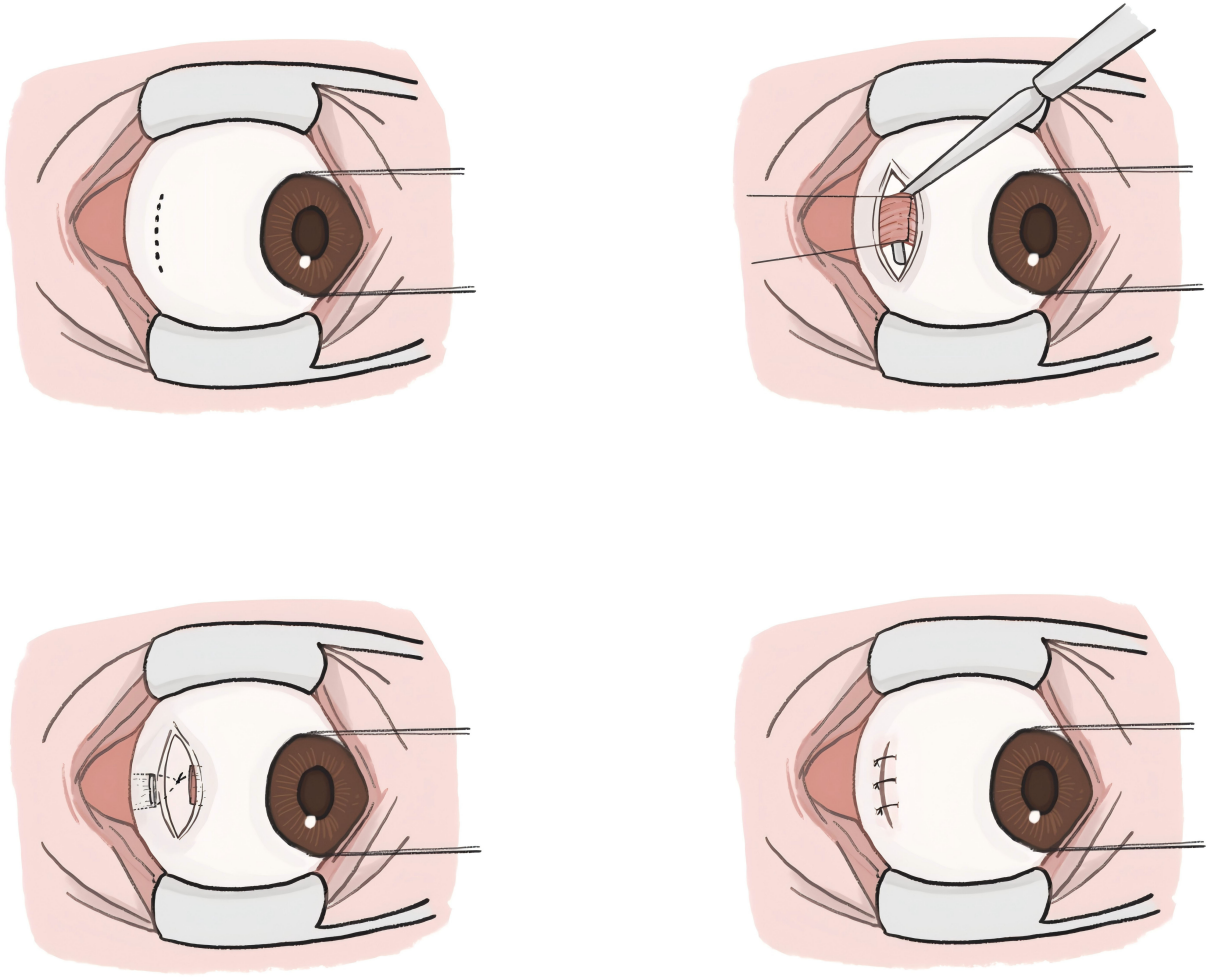
Schematic diagram of the modified swan incision (MSI) combined with lateral rectus suspension.

**FIGURE 3:**
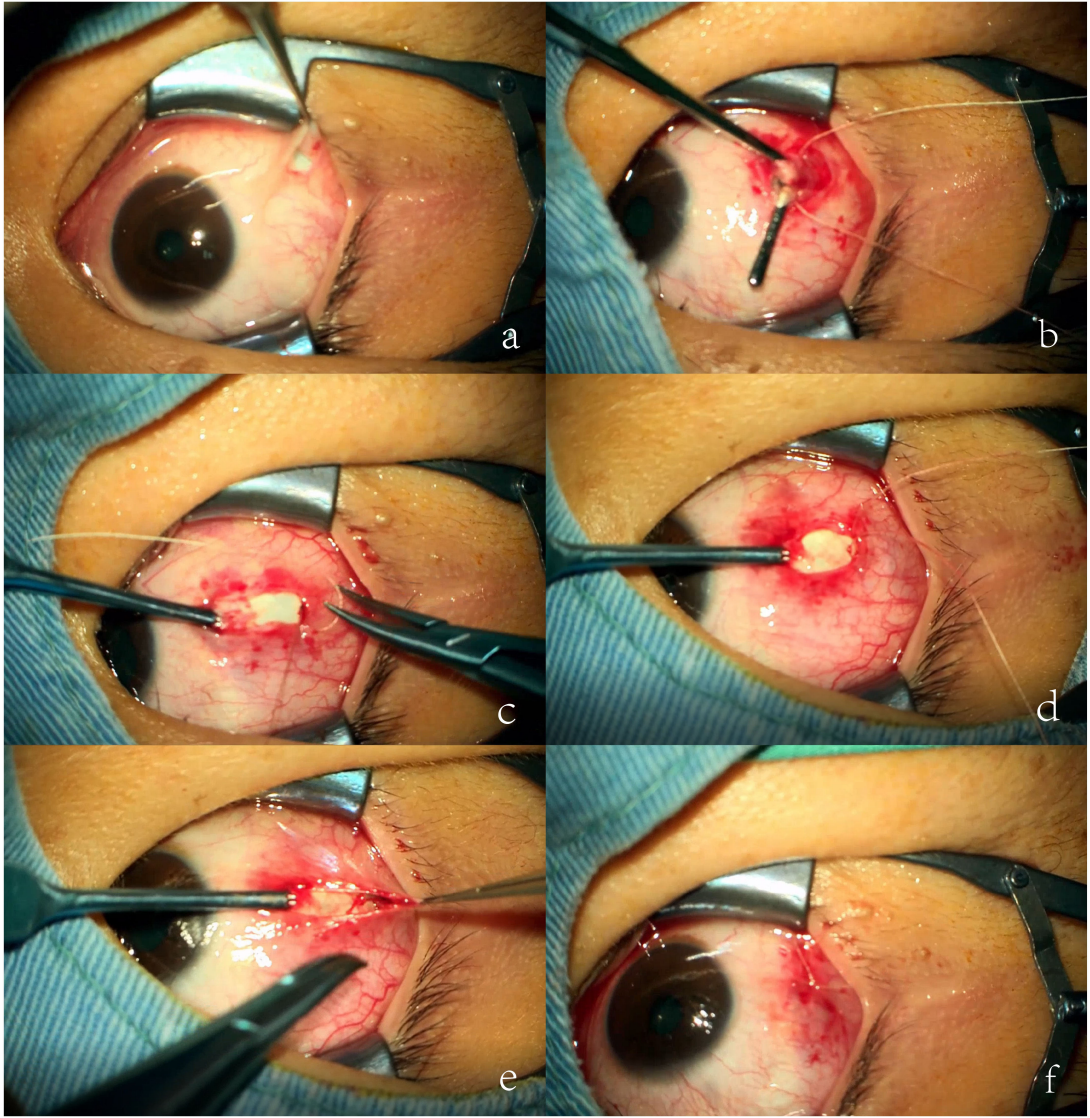
Actual picture of the modified swan incision (MSI) combined with lateral rectus suspension. **(a)** Making an MSI incision in the temporal conjunctiva. **(b)** Separating the lateral rectus from the surrounding fascia to expose the rectus. Before cutting the tendon, a set of loop sutures is pre-placed on each side 1 mm posterior to the muscle insertion. **(c)** The lateral rectus is then cut at its insertion point. **(d)** Approximately 5 mm posterior to the original insertion, the sutures are passed parallel to borders of the lateral rectus through the superficial sclera. The distance between the new muscle insertion and the planned suture ligation point is measured to confirm alignment with the preoperative recession amount. **(e)** Once confirmed, the sutures are tied, forming a hammock-like structure. **(f)** The conjunctival incision is closed.

**FIGURE 4:**
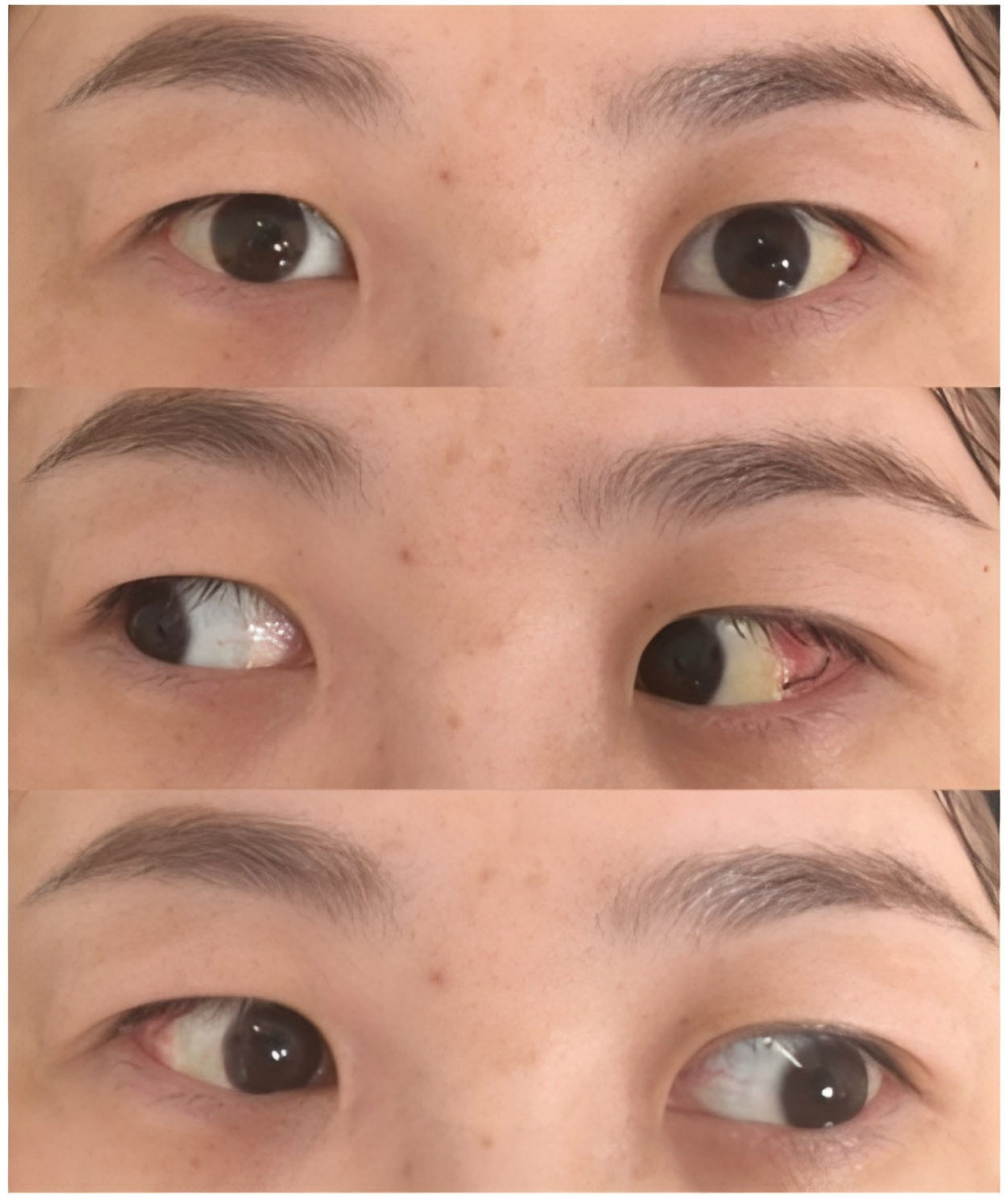
Postoperative eye photo of the patient 1 week after undergoing MSI combined with bilateral lateral rectus suspension.

Although the reduced incision size may limit the surgical field of view and increase the difficulty of performing traditional lateral rectus recession, this issue is effectively addressed by adopting the lateral rectus suspension. Instead of directly suturing the muscle to a new insertion point, the muscle is suspended to a pre-measured position (12). This method aligns well with MSI, meeting the requirements of minimally invasive surgery while being suitable for treating large-angle exotropia, as illustrated in Figures 2, 3. Figure 4 is an image of a patient 1 week after undergoing MSI combined with bilateral lateral rectus suspension. It clearly shows that the MSI incision is very discreet when the eye is in primary position.

Moreover, for medial rectus muscle resection and recession amount within the 6 mm, the MSI provides adequate surgical field exposure to safely perform these operations. Currently, the MSI technique is clinically applicable only for the correction of horizontal strabismus (including both esotropia and exotropia) and is not yet indicated for cases involving vertical strabismus.

## 2 Materials and methods

This study was conducted in accordance with the Declaration of Helsinki and approved by the Institutional Review Board and local Ethics Committee of the Affiliated Drum Tower Hospital, Medical School of Nanjing University (Protocol code: 2025003601).

This study is a retrospective analysis aimed at accessing the efficacy of the MSI technique in strabismus surgery. As a retrospective cohort study, although randomization was not employed, the comparability between the groups was ensured through the use of uniform inclusion/exclusion criteria and a baseline balance analysis.

**FIGURE 5:**
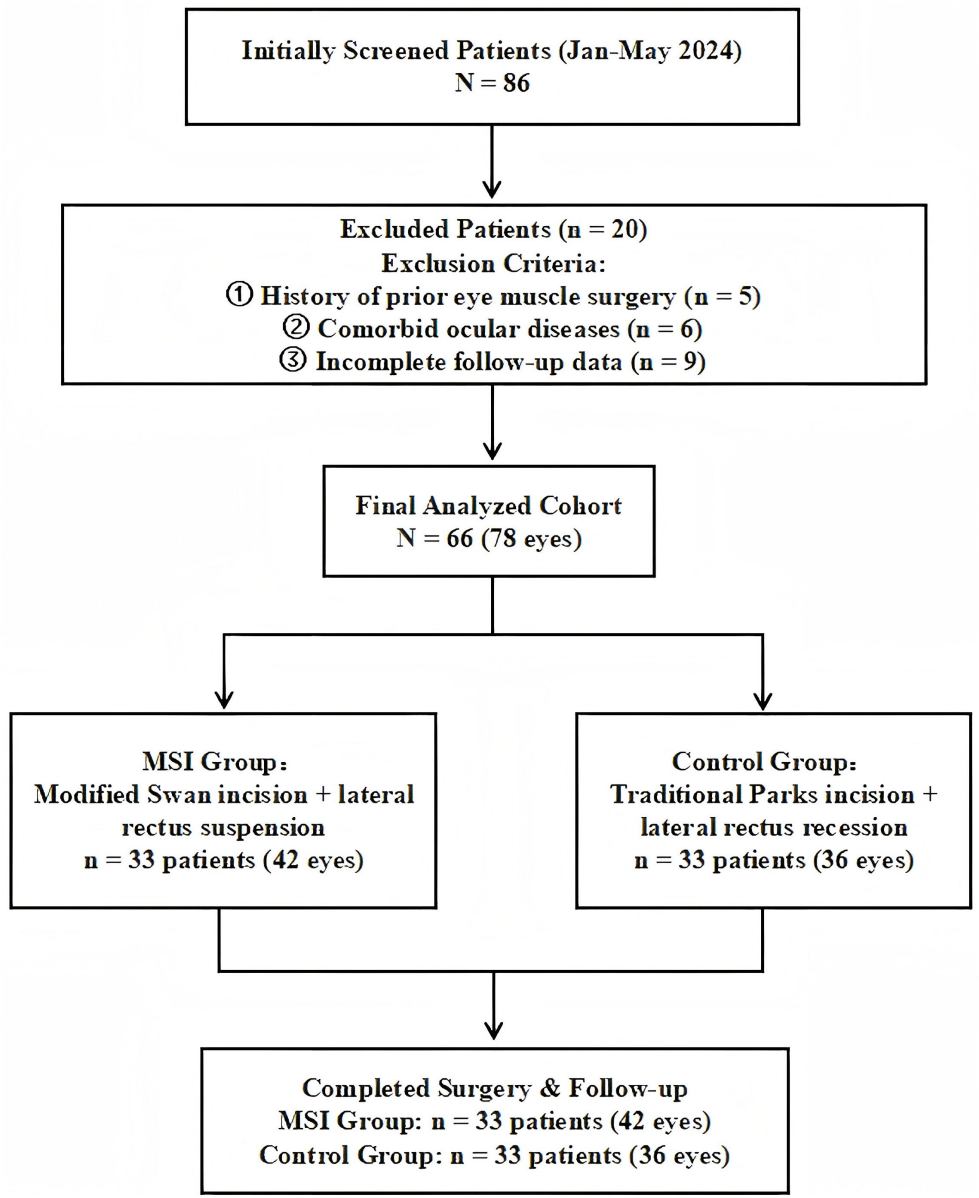
Research flowchart: patient enrollment, exclusion, and grouping process.

We retrospectively collected the historical examination results and surgical records of 86 patients who underwent strabismus surgery from January 2024 to May 2024. Inclusion criteria: (1) Subjects aged between 5 and 70 years; (2) Subjects diagnosed with exotropia through ophthalmic examination and undergoing strabismus surgery. Exclusion criteria: (1) History of previous extraocular muscle surgery; (2) Comorbidities such as glaucoma, severe dry eye syndrome, or other ocular conditions that may affect surgical evaluation; (3) Postoperative follow-up of less than 6 months or significant missing clinical data within 6 months post-surgery. The study finally included 66 (78 eyes) patients. The flowchart is shown in Figure 5.

The selection of surgical approach was determined through shared decision-making between surgeons and patients. The MSI technique, as an innovative approach, offers advantages such as a smaller incision size and more concealed scarring. However, as a newly developed surgery, its long-term efficacy data are still being accumulated. In contrast, the conventional technique is supported by well-established evidence-based medicine with proven and stable corrective outcomes, though it requires larger conjunctival incisions that may lead to more pronounced postoperative inflammatory responses and visible scarring. After patients express their preferences, the surgical team conducts a secondary assessment to verify the suitability of the selected technique based on surgical indications, ultimately determining the individualized surgical plan.

All surgeries is performed under topical combine with local anesthesia, using obucaine hydrochloride eye drops and subconjunctival injection of 2% lidocaine. The modified Swan incision was used for the MSI group and the Parks incision for the control group. In control group, for patients undergoing surgery on one-muscle, unilateral lateral rectus (LR) recession or unilateral medial rectus (MR) resection was performed; for patients with two-muscles involved, bilateral lateral rectus recession (BLRc) or unilateral lateral rectus recession combined with medial rectus resection (R&R) was used. All surgeries in this study were performed by the same experienced surgeon to ensure consistency in surgical technique.

In the MSI group, for patients undergoing surgery on one muscle, unilateral LR suspension was performed; for patients with two muscles, bilateral LR suspension or unilateral LR suspension combined with MR resection was performed.

For patients with three muscles, both groups included two patients each. The Control group underwent BLRc combined with unilateral MR resection, while the MSI group underwent bilateral LR suspension combined with unilateral MR resection.

In control group, for patients undergoing surgery on one-muscle, unilateral lateral rectus (LR) recession or unilateral medial rectus (MR) resection was performed; for patients with two-muscles involved, bilateral lateral rectus recession (BLRc) or unilateral lateral rectus recession combined with medial rectus resection (R&R) was used.

In the MSI group, for patients undergoing surgery on one muscle, unilateral LR suspension was performed; for patients with two muscles, bilateral LR suspension or unilateral LR suspension combined with MR resection was performed.

All of the above surgeries including LR recession, LR suspension, and MR resection, were performed using the traditional Parks incision in Control group and the modified Swan incision in MSI group. The specific surgical procedures for patients with a deviation greater than 40 PD are shown in Table 1.

**TABLE 1:** Surgical procedures for patients with deviation greater than 40PD.

Group & deviation	Specific surgical procedures	Number of patients
MSI group	BLR suspension	*N* = 3
Deviation ≥ 40 PD	LR suspension & MR resection	*N* = 6
	BLR suspension & MR resection	*N* = 2
	LR suspension	*N* = 2
Control Group	R&R	*N* = 14
Deviation ≥ 40 PD	BLRc & MR resection	*N* = 2
	MR resection	*N* = 1

BLR suspension, bilateral lateral rectus suspension; LR suspension, unilateral lateral rectus suspension; MR resection, unilateral medial rectus resection; R&R, unilateral lateral rectus recession combined with medial rectus resection; BLRc, bilateral lateral rectus recession.

For MSI combined with lateral rectus suspension, an incision is first made about 10 mm posterior to the temporal corneal margin, perpendicular to the LR, approximately 5 mm in length. The next LR suspension steps are as follows: (1) Separate the LR from the surrounding fascia to expose the rectus; (2) Before cutting the tendon, a set of 6-0 polyester loop sutures is pre-placed about 1 mm posterior to the muscle insertion on both sides of the LR in preparation for suspension; (3) The LR is then cut at its insertion point; (4) Approximately 5 mm posterior to the original muscle insertion, the suture is passed through the superficial sclera; (5) Measure the distance between the new muscle insertion and the planned suture ligation point to ensure alignment with the preoperative recession amount; (6) Once confirmed, tie the sutures to form a hammock-like structure; (7) Finally, suture the conjunctival incision with 7-0 sutures. As shown in Figures 3, 4.

It is important to note that at the new muscle insertion, the muscle is not sutured to the sclera as in LR recession, but instead rest against the sclera surface by the suspension sutures placed approximately 5 mm posterior to the original muscle insertion point.

In both groups, the conjunctiva was sutured with absorbable 7-0 sutures. Dexamethasone Ophthalmic Ointment was applied to the conjunctival sac of lower eyelid and the surgical eye was bandaged with gauze. The patient received levofloxacin eye drops for 1 week after surgery.

This study evaluates the surgical outcomes through multiple indicators, primarily including eye position and surgical success rate, postoperative inflammatory response, changes in visual acuity and diplopia, as well as comparisons of surgical time. To enhance result reliability, all postoperative evaluations were conducted independently by clinicians not involved in the surgical procedures. Furthermore, assessment validity was strengthened through dual evaluations, with a randomly selected subset of cases examined independently by multiple physicians to verify consistency.

In assessing postoperative eye position, the degree of strabismus is measured at 1 week, 3 months, and 6 months post-surgery to evaluate the stability and long-term effectiveness of the surgery in correcting eye alignment. Based on clinical experience and previous literature, surgical success is defined as achieving orthophoria or proper correction (exotropia less than 10 PD and esotropia less than 5 PD) at 6 months post-surgery, along with the restoration of binocular fusion function (13).

Postoperative inflammatory Response mainly include the Redness Score and the Foreign Body Sensation Score (FBS Score). The Redness Score evaluates conjunctival hyperemia in four quadrants (superotemporal, inferotemporal, superonasal, inferonasal), graded from 0 (no hyperemia) to 3 (severe hyperemia) based on the extent and severity of congestion. The FBS Score reflects the degree of postoperative ocular discomfort, ranging from 0 (no sensation) to 3 (severe sensation), assessing levels from mild discomfort to severe impairment of daily life, potentially requiring medication for relief. Monitoring these inflammatory response offers a direct assessment of early postoperative inflammatory situation, reflecting both tissue damage severity and initial recovery progression.

Statistical analyses were performed using IBM SPSS Statistics 27.0. The normality of continuous variables was assessed using the Shapiro-Wilk test. Based on the test results, data are presented as mean (standard deviation) or median (interquartile range).

This study employed generalized estimating equations (GEE) to analyze the following outcome measures: (1) orthocorrection rates at different time points; (2) Redness Score and Foreign Body Sensation (FBS) Score measured at the eye level.

Based on the type of variables, a binary logistic regression model was used for the surgical success rates at 6 months, reduced vision rate, and diplopia rate between two groups.

Although the Shapiro-Wilk test did not reject the assumption of normality for surgical time in both groups (MSI group *P* = 0.126; Control group *P* = 0.807), we opted for a Generalized Linear Model (Gamma distribution with log link) for a more robust analysis. This decision was based on the theoretical expectation that such time variables are typically skewed, coupled with the concern that the statistical power of the normality test might be insufficient to detect a deviation from normality given the current sample size.

All models were adjusted for covariates including age, deviation, operated eye(s) (unilateral/bilateral), and number of muscles involved in surgery to control for potential confounding effects.

To control for inflation of type I error due to multiple comparisons, the *P* values for all secondary endpoints were adjusted using the False Discovery Rate (FDR) method. The adjusted *P* values are reported as *q* values, and a *q* < 0.05 was considered statistically significant. The *P* value for the primary endpoint (redness score) was not adjusted.

## 3 Results

### 3.1 General information of two groups

This study included 66 patients with exotropia who underwent surgical treatment. The MSI group comprised 33 patients (42 eyes), and the control group included 33 patients (36 eyes). The specific baseline data for both groups are presented in Table 2.

**TABLE 2:** Comparison of baseline data between two groups.

Characteristics	MSI group	Control group	*P*-value	SMD
Number of patients	33 patients(42 eyes)	33 patients(36 eyes)		
Sex				
Female	22	17		
Male	11	16		
Age				
Median (IQR)	28 (19-41)	26 (18–41)	0.457	0.132
Range	[11, 63]	[9, 63]		
Deviation (Prism diopter, PD)				
Median (IQR)	35 (25–60)	45 (25–60)	0.364	0.212
Range	[20, 100]	[20, 100]		
Numbers of muscle, mean ± SD	1.85 ± 0.51	1.94 ± 0.43	0.435	0.212
Unilateral vs. bilateral, *n* (%)			0.741	
Unilateral	27 (81.82)	28 (84.85)		0.085
Bilateral	6 (18.18)	5 (15.15)		−0.085

The Mann-Whitney U test results showed no statistically significant differences between the two groups in terms of age (*P* = 0.457) or deviation (*P* = 0.364).

To more comprehensively assess balance, standardized mean differences (SMD) were calculated. The SMD values for age and deviation were 0.137 and 0.192, respectively. According to Cohen’s criteria (SMD < 0.2 indicates a small effect size), this suggests that the actual effect size of the baseline differences between the two groups was small. In conclusion, it can be considered that the baseline data of the two groups were balanced before surgery, demonstrating good comparability.

### 3.2 Comparison of surgical time and correction efficacy between the two groups

Univariate analysis revealed that the MSI group had a significantly shorter operative time compared to the Control group (39.97 ± 15.13 min vs. 71.70 ± 16.32 min, *P* < 0.0001). A generalized linear model (Gamma distribution with log link function) was employed for multivariable analysis to adjust for the potential confounding effects of surgical complexity (number of muscles, unilateral/bilateral).

The overall model was statistically significant (likelihood ratioχ^2^ = 49.073, *P* < 0.001). After adjusting for the number of muscles and unilateral/bilateral factors, the surgical approach group remained an independent and significant predictor of operative time (Waldχ^2^ = 26.088, *P* < 0.001). The adjusted time ratio for the MSI technique was 0.504 (95% CI: 0.388–0.656), indicating that, compared to the conventional technique, the MSI technique reduced the mean operative time by approximately 49.6%.

The estimated marginal means derived from the model showed that, after adjusting for complexity, the mean operative time for the MSI group was 39.0 min (95% CI: 33.2–45.7 min), significantly lower than that of the control group at 77.3 min (95% CI: 64.0–93.2 min), with a mean difference of -38.3 min (Bonferroni-corrected *P* < 0.001).

In the MSI group, the rates of proper correction at 1 week, 3 months, and 6 months post-surgery were 93.94% (31 cases), 81.24% (27 cases), and 75.76% (25 cases), respectively. In the control group, the corresponding rates were 81.82% (27 cases), 72.73% (24 cases), and 72.73% (24 cases).

The results indicated that there was no significant difference in the rates of proper correction between two groups (OR = 1.17, 95% CI 0.39–3.54, *P* = 0.778). Regarding the time effect, the rate of proper correction at 1 week showed a tendency to be higher than at 6 months (OR = 1.69, 95% CI 0.95–2.99, *P* = 0.072), though this did not reach statistical significance. No significant difference was observed between 3 months and 6 months (*P* = 1.000).

The group-by-time interaction was not statistically significant (1 week, *P* = 0.133; 3 months, *P* = 0.147), suggesting that the changes in treatment efficacy over time followed similar trends in both groups.

One patient in each group achieved orthophoria at 6 months post-surgery but was not considered a successful correction due to persistent binocular diplopia. The surgical success rates were 72.72% (24 cases) in the MSI group and 69.70% (23 cases) in the control group. Binary logistic regression analysis revealed no statistically significant difference in surgical success rates between the MSI group and the control group (OR = 0.67, 95% CI: 0.25–1.78, *P* = 0.422).

### 3.3 Comparison of postoperative inflammatory response and visual changes between the two groups

The median Redness score was 1 [IQR: 1–2] in the MSI group and 2 [IQR: 1.25–2] in the control group. GEE analysis showed that after adjusting for age, strabismus angle, unilateral/bilateral surgery, and number of muscles operated, the MSI group had significantly lower redness score compared to the control group (OR = 0.117, 95% CI: 0.042–0.330, *P* < 0.001). No statistically significant difference was found in FBS score between the two groups (OR = 0.465, 95% CI: 0.181–1.191, *P* = 0.110).

No significant association was observed between the type of surgery and postoperative vision decline (OR = 0.84, 95% CI: 0.24–2.90, *P* = 0.781). There was no significant difference in the incidence of postoperative diplopia between the two surgical approaches (OR = 0.89, 95% CI: 0.18–4.42, *P* = 0.887).

The redness score at 1 week was designated as the primary endpoint of this study, with all other endpoints listed as secondary. For all secondary endpoints, we have applied FDR correction for multiple comparisons and have reported the corresponding *q*-values. The corrected *q* values for all variables are presented in Tables 3, 4. After FDR correction, statistical analysis revealed that only surgical time showed a significant difference between groups (*q* = 0.010), while no statistically significant differences were observed in the other indicators.

**TABLE 3:** Comparison of primary and secondary endpoints between the MSI and control groups.

Variable	MSI Group (*n* = 33 patients/42 eyes)	Control Group (*n* = 33 patients/36 eyes)	Statistical test	Effect size (95% CI)	*P* value	*q* value
**Primary endpoint**						
Redness score at 1 w (per-eye), median (IQR)	1 (1–2)	2 (1.25–2)	Generalized Estimating Equations	[0.04, 0.33]	<0.001	–
**Secondary endpoints**						
Surgical time (per-patient), mean ± SD, min	39.97 ± 15.13	71.70 ± 16.32	A generalized linear model	[−54.8, −21.7]	<0.001	0.050
FBS score at 1 w (per-eye), median (IQR)	0 (0–1)	1 (0–1)	Generalized Estimating Equations	[0.18, 1.19]	0.110	0.275
Orthotropic Rate (per-patient) at 1w, *n* (%)	31 (93.94%)	27 (81.82%)		–	–	–
Orthotropic Rate (per-patient) at 3 m, *n* (%)	27 (81.24%)	24 (72.73%)				
Orthotropic Rate (per-patient) at 6 m, *n* (%)	25 (75.76%)	24 (72.73%)				
Surgical success (per-patient), *n* (%)	24 (72.72%)	23 (69.70%)	logistic regression	[0.25,1.78]	0.422	0.703
Reduced vision (per-patient), *n* (%)	2 (6.06%)	4 (12.12%)		[0.24, 2.90]	0.781	0.887
Diplopia (per-patient), *n* (%)	1 (3.03%)	2 (6.06%)		[0.18, 4.42]	0.887	0.887

CI, Confidence Interval; “-” indicates that the metric is not applicable for that row.

**TABLE 4:** Analysis of orthotropic rates using GEE.

Effect	Comparison	OR (95% CI)	*P* value	*q* value
**Group effect**				
	MSI vs. Control	1.17 (0.39– 3.54)	0.778	0.786
**Time effect**				
	1 Week vs. 6 Months (Ref.)	1.69 (0.95 –2.99)	0.072	0.118
	3 Months vs. 6 Months (Ref.)	**–**	1.000	1.000
Interaction effect group * time				
	At 1 week	**–**	0.133	0.222
	At 3 Months	**–**	0.147	0.222

OR, odds ratio; CI, confidence interval; GEE, generalized estimating equations; “-” indicates that the metric is not applicable for that row.

## 4 Discussion

This study utilized a complete-case (per-protocol) analysis, which included only patients with a minimum of 6 months of follow-up.

The three traditional incisions commonly used in strabismus surgery are the limbal incision, Parks incision, and Swan incision. Each of these approaches has distinct advantages and limitations (4). The limbal incision provides extensive conjunctival dissection, facilitating a broad surgical field and greater maneuverability for the surgeon (14). However, its large incision size increases the risk of infection and may result in noticeable postoperative scarring, which can negatively impact cosmetic outcomes. The Parks incision is placed in the concealed fornix, offering the benefit of hidden scars beneath the eyelid for better aesthetics (15). However, it is technically more challenging, which limited surgical exposure that increase the procedural complexity. The Swan incision, typically measuring 10–12 mm in length, refers to a incision made at the muscle insertion (6). It provides straightforward access and clear field but carries disadvantages of a large incision and potential risk of muscle injury.

The fundamental surgical techniques in strabismus include recession, resection, and adjustable sutures (16). For exotropia cases, commonly employed methods include BLRc, R&R, and BLRc with augmentation (17, 18). In cases requiring larger recessions, lateral rectus suspension can be used as an alternative to the conventional lateral rectus recession, where the muscle is sutured directly to the new insertion.

Although traditional incisions remain widely utilized, they present limitations such as large wound sizes, visible postoperative scarring, and increased procedural complexity. The lateral rectus suspension is typically performed with limbal incision (12), which results in a relatively large wound. To address these challenges, we propose a modified Swan incision (MSI) combined with lateral rectus suspension for the treatment of exotropia.

First, the MSI group had a advantage in surgical time. We employed a GLM with a Gamma distribution and log-link function, controlling for the number of muscles operated on and whether the procedure was unilateral or bilateral. This analysis confirmed that the MSI technique remained a significant independent factor associated with reduced operative time. Research by Chen’s group has established that the suspension saves time compared to traditional recession (19). Our study attributes this phenomenon to recession requires suturing both the muscle and the sclera, which demands precise control of placement and suture thickness, and both ends of the muscle need to be sutured. In contrast, the suspension simplifies the process as it only involves passing the suture itself through the superficial sclera and tying a knot, thus saving time. It is important to note, however, that the surgeon’s greater familiarity with the MSI technique compared to the Parks incision may have also influenced operative times–a potential confounding factor acknowledged in the study limitations.

Next, the comparison of postoperative inflammatory response further highlighted the benefits of MSI. The small incision and short surgery time of MSI technique help reduce inflammation. Although there was no significant difference in FBS score between the two groups, patients in the MSI group recovered more quickly, with most reporting mild or no foreign body sensation at 1 week, suggesting an improvement in postoperative ocular comfort due to the MSI.

It should be noted that our study employed relatively stringent criteria for surgical success, requiring not only orthophoria (exotropia less than 10 PD and esotropia less than 5 PD) at 6 month but also binocular fusion function. In fact, when evaluated solely based on ocular alignment criteria, our results are essentially consistent with the long-term outcomes of rectus suspension techniques reported by Rodrigues (20). Meanwhile, our study included a certain proportion of patients with intermittent exotropia. According to research by Ekdawi, such patients tend to have higher postoperative recurrence rates, with success rates potentially declining to 46% at 5 years (21), which may represent an important reason for the observed decrease in success rates during follow-up.

In both groups, we performed meticulous dissection of rectus, avoiding excessive traction or compression of the tissues, ensuring that the tissues around the incision site did not come into contact, thus guaranteeing sufficient exposure of rectus. Postoperatively, we used antibiotic eye drops for the patients and scheduled regular follow-up visits. These may help reduce the incidence of adhesions.

At present, the MSI technique is only used for the correction of horizontal strabismus, and its applicability to cases involving vertical strabismus requires further investigation. In the future, we plan to improve the MSI technique by modifying the incision position to approach the fornix, which will allow for its use in other types of strabismus surgeries. As this study is a retrospective research, we regret that we were unable to obtain retrospective data on the traditional Swan incision for direct comparison, which constitutes a limitation of our study design. Due to the retrospective nature of this study, inter-rater reliability for the redness and FBS score could not be quantitatively assessed.

In conclusion, the modified Swan incision combined with lateral rectus suspension significantly shortens surgical time, reduces postoperative inflammation, and optimizes postoperative recovery, making it highly valuable for clinical application. Future research should further expand sample sizes and conduct prospective studies to assess its long-term efficacy and impact on patients’ quality of life.

## 5 Conclusion

Compared to traditional techniques, the modified Swan incision (MSI) combined with lateral rectus suspension significantly reduces surgical time, enhances postoperative satisfaction, and offers advantages such as reduced postoperative inflammation and faster recovery, demonstrating high clinical value. MSI combined with lateral rectus suspension is suitable for patients with exotropia, particularly in strabismus correction where minimally invasive approaches and aesthetic considerations are essential.

## Data Availability

The raw data supporting the conclusions of this article will be made available by the authors, without undue reservation.
